# Pathology of US Porcine Epidemic Diarrhea Virus Strain PC21A in Gnotobiotic Pigs

**DOI:** 10.3201/eid2004.131685

**Published:** 2014-04

**Authors:** Kwonil Jung, Qiuhong Wang, Kelly A. Scheuer, Zhongyan Lu, Yan Zhang, Linda J. Saif

**Affiliations:** The Ohio State University, Wooster, Ohio, USA (K. Jung, Q. Wang, K.A. Scheuer, Z. Lu, L.J. Saif);; Ohio Department of Agriculture, Reynoldsburg, Ohio, USA (Y. Zhang)

**Keywords:** porcine epidemic diarrhea virus, porcine epidemic diarrhea, pathogenesis, outbreak, USA, gnotobiotic pigs, US strain, pigs, strain PC21A, viruses, enteropathogenic

## Abstract

To understand the progression of porcine epidemic diarrhea virus infection, we inoculated gnotobiotic pigs with a newly emerged US strain, PC21A, of the virus. At 24–48 hours postinoculation, the pigs exhibited severe diarrhea and vomiting, fecal shedding, viremia, and severe atrophic enteritis. These findings confirm that strain PC21A is highly enteropathogenic.

A highly contagious coronavirus that causes porcine epidemic diarrhea (PED) was first reported in the United States in May 2013 in Iowa. Since then, the virus—porcine epidemic diarrhea virus (PEDV)—has spread rapidly nationwide (*1*,*2*). PEDV (family *Coronaviridae,* genus *Alphacoronavirus*) was previously reported only in Europe and Asia. The first US outbreaks caused a high number of deaths among suckling pigs and, as a consequence, substantial economic losses (*1*,*2*).

Results of PEDV pathogenesis studies using the prototype European PEDV strain, CV777, were reported in the 1980s (*3*,*4*). Strain CV777 infections caused intestinal villous atrophy with substantially reduced ratios of villous height to crypt depth (VH:CD) (*3*,*4*). Pathogenic features of CV777 are similar to those observed for Asian PEDV strains that circulated in the 1990s (*4*–*6*). To understand the progression of PEDV infection, we studied the pathogenesis of the newly emerged US strain, PC21A. 

## The Study

**Figure 1:**
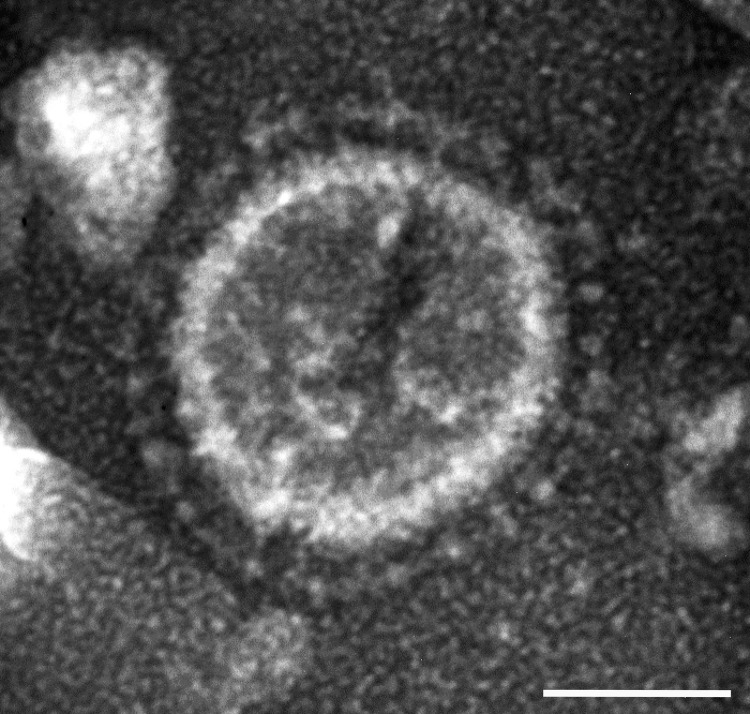
Electron micrograph of a US porcine epidemic diarrhea virus (PEDV) particle detected in a field fecal sample collected during a 2013 outbreak of PED on a farm in Ohio, USA; the fecal sample from which PEDV strain PC21A in this study was obtained was from a pig on the same farm during the same outbreak. The sample was negatively stained with 3% phosphotungstic acid. Scale bar = 50 nm.

In June 2013, intestinal contents were obtained from a 1-day-old pig with diarrhea on a farm in Ohio, USA. PEDV strain PC21A was detected in the sample by reverse transcription PCR (RT-PCR) selective for the nucleocapsid gene (229–557 nt). The partial nucleocapsid gene sequence of PC21A was identical to that of 2 US PEDV outbreak strains from Colorado, USA: USA/Colorado/2013 (GenBank accession no. KF272920) and 13-019349 (GenBank accession no. KF267450). Only coronavirus-like particles were observed in the fecal sample by electron microscopy (Figure 1). The sample was negative for rotavirus groups A and C and for transmissible gastroenteritis virus/porcine respiratory coronavirus by RT-PCR (*7*,*8*). 

The sample was bacteriologically sterilized by using 0.22-μm syringe filters and then prepared as inoculum. Near-term gnotobiotic pigs were delivered aseptically by hysterectomy from a specific pathogen–free sow (*9*). Six 10- to 35-day-old pigs were randomly assigned to a PEDV-infected group (pigs 1–5) or a negative control group (pig 6). Information about inoculation and inocula pig-passage number is described in Table 1. Pigs 1–3 and 5 were inoculated orally and/or intranasally with 6.3–9.0 log_10_ genomic equivalents (GE) of PEDV strain PC21A; pig 4 was exposed to the virus by indirect contact with inoculated pig 3. For each sample, the quantity of PEDV RNA GE was ≈10^6^ times higher than plaque assay results for a cell-adapted PEDV strain, PC22A. Clinical signs were monitored hourly. Pig 4 was monitored for longer-term clinical signs and virus shedding. Pigs were euthanized for pathologic examination at 3 stages of infection: acute, mid, and later stages (<24 h, 24–48 h, and >48 h, respectively, after onset of clinical signs). The Ohio State University Institutional Animal Care and Use Committee approved all animal-related experimental protocols.

**Table 1:** Design and results of a study of the pathology of US PEDV strain PC21A in gnotobiotic pigs, 2013*

Pig status, pig no.; age, d, at inoculation	Inoculum passage no.	Intranasal/oral inoculum, log_10_ GE						Onset of clinical signs, PIH	Viral RNA in serum sample at euthanasia, log_10_ GE/mL†
			Fecal shedding, log_10_ GE/mL, by PIH						
			0	24	48	72	96		
PEDV-inoculated									
1; 10	1	7.3/7.7	<4.8†	10.7‡	–	–	–	25–26	7.6
2; 18	2	6.3/6.8	<4.8	<4.8	11.0	11.2‡	–	44–46	6.3
3; 24	3	8.3/8.8	<4.8	10.2	12.3‡	–	–	44–46	5.7
4; 24§	4	Indirect contact	<4.8	10.9	9.6	10.6	ND	2–4§	7.6
5; 35	4	0/9.0	<4.8	ND	ND	–	–	26–30	4.8
Negative control									
6; 26	.	.	<4.8	<4.8	<4.8	<4.8	<4.8	.	<3.8†

*PEDV, porcine epidemic diarrhea virus; GE, genome equivalents; PIH, postinoculation hour; –, no result (pig euthanized); ND, not determined. †Detected by real-time reverse transcription PCR with a detection limit of 4.8 log_10_ GE/mL for fecal samples and 3.8 log_10_ GE/mL for serum samples.  ‡Euthanized. §At 24 days of age, noninoculated pig 4 was exposed by indirect contact to pig 3 (at PIH 0) through small holes drilled into the stainless steel divider panel located between the 2 pigs in the shared pig tub isolator unit. Clinical signs and virus shedding were monitored after indirect contact. Diarrhea and vomiting developed in pig 4 approximately 2–4 h after clinical signs developed in pig 3 (i.e., in pig 4, signs developed 46–50 h after indirect contact with inoculated pig 3); pig 4 was euthanized ≈120 h after the onset of clinical signs.

Fecal or rectal swab samples were prepared as described (*9*). Virus RNA was extracted by using the MagMAX Viral RNA Isolation Kit (Applied Biosystems, Foster City, CA, USA) according to the manufacturer’s instructions. Titers of virus shed in feces were determined by TaqMan real-time RT-PCR using the OneStep RT-PCR Kit (QIAGEN, Valencia, CA, USA) as reported (*10*), with modifications in the forward primer and probe to provide a 100% match to the US strains: forward 5′-CGCAAAGACTGAACCCACTAAC-3′ and probe FAM-TGYYACCAYYACCACGACTCCTGC-BHQ. A standard curve was generated by using the PCR amplicon (PEDN 229/557) of strain PC21A. The detection limit was 10 GE per reaction, corresponding to 4.8 log_10_ and 3.8 log_10_ GE/mL of fecal and serum samples, respectively.

Small and large intestine tissues, lung, liver, heart, kidney, spleen, and mesenteric lymph node were examined grossly and histologically. Mean jejunal VH:CD was measured by using PAX-it software (PAXcam, Villa Park, IL, USA) as described (*11*). The frozen tissues were prepared and tested by immunofluorescence staining, as described (*12*), for the detection of PEDV antigen, using monoclonal antibody 6C8-1 against the spike protein of PEDV strain DR13 (provided by Daesub Song, Korea Research Institute of Bioscience and Biotechnology, Daejeon, Korea).

Acute, severe watery diarrhea and vomiting developed in all inoculated pigs. Clinical signs developed 24–48 h after inoculation, regardless of the inoculum dose or number of inoculum pig passages (Table 1). Pig 4, which was followed longer, also exhibited dehydration, loss of bodyweight, and lethargy, but it consumed most of the milk that was offered. However, ≈120 h after onset of clinical signs, pig 4 collapsed after showing signs of disorientation and emaciation. 

Immune electron microscopy, using a gnotobiotic pig hyperimmune serum to PEDV, showed only PEDV particles in the intestinal contents. For the pig-passaged PC21A strain, RT-PCR/PCR results were negative for transmissible gastroenteritis virus/porcine respiratory coronavirus (*7*), rotavirus groups A–C (*8*), caliciviruses (*13*,*14*), astroviruses (*15*), circoviruses, enterovirus, kobuvirus, and bocavirus. For pigs 1 and 2, the detection of fecal virus shedding 24–48 h after inoculation coincided with the onset of clinical signs; for pigs 3 and 4, fecal shedding occurred before the onset of clinical signs (Table 1).

**Figure 2:**
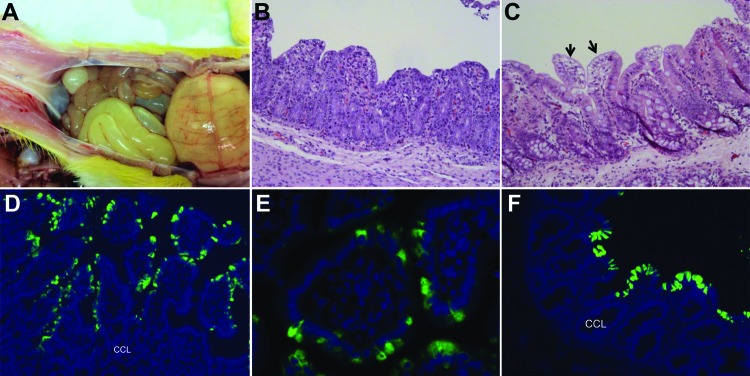
Changes seen, by macroscopic examination, histologic examination, or immunofluorescence staining in the intestine of gnotobiotic pigs inoculated with porcine epidemic diarrhea virus (PEDV; US strain PC21A). A) Intestine of pig 1 at postinoculation hour (PIH) 30 (4–5 h after onset of clinical signs), showing thin and transparent intestinal walls (duodenum to colon) and extended stomach filled with curdled milk. B) Hematoxylin and eosin (H&E)–stained jejunum of pig 3 at PIH 46 (at onset of clinical signs), showing acute diffuse, severe atrophic jejunitis. Original magnification ×200. C) H&E-stained cecum of noninoculated pig 4 (which was exposed to inoculated pig 3 at PIH 0) at 120 h after onset of clinical signs. Acute diffuse, mild vacuolation of superficial epithelial cells (arrows) and subepithelial edema are seen. Original magnification ×200. D) Immunofluorescence staining of jejunum of pig 5 at PIH 67 (37–41 h after onset of clinical signs), indicating that the epithelial cells lining atrophied villi are positive for PEDV. Original magnification ×200. E) Immunofluorescence staining of jejunum of pig 3 at PIH 46 (at onset of clinical signs), showing localization of PEDV antigens in the cytoplasm of enterocytes. Original magnification ×600. F) Immunofluorescence staining of colon of pig 2 at PIH 72 (26–28 h after onset of clinical signs), showing large numbers of PEDV-positive cells. Original magnification ×200. CCL, crypt cell layer. Nuclei were stained with blue-fluorescent 4′, 6-diamidino-2-phenylindole, dihydrochloride.

By macroscopic examination, all infected pigs exhibited typical PEDV-like lesions, characterized by thin and transparent intestinal walls (duodenum to colon) and accumulation of large amounts of yellowish fluid in the intestinal lumen (Figure 2, panel A). The stomach was filled with curdled milk, possibly due to reduced intestinal peristalsis. The other internal organs appeared normal. Histologic lesions included acute diffuse, severe atrophic jejunitis (Figure 2, panel B) and mild vacuolation of superficial epithelial cells and subepithelial edema in cecum and colon (Figure 2, panel C). These findings were similar to those in conventional pigs naturally infected with Asian or US strains of PEDV and in caesarean-derived, colostrum-deprived pigs experimentally infected with CV777 (*2*,*3*,*5*,*6*). The mean jejunal VH:CD of the 5 infected pigs ranged from 1.2 to 3.4, probably depending on the stage of infection (Table 1), and that of the negative control pig was 6.3 (±0.2). VH:CD for pig 4, which was euthanized at a later stage of infection, was 1.5 (±0.2), a ratio indicative of continued cellular necrosis. Neither clinical signs nor lesions developed in the negative control pig during the experiment.

Immunofluorescence-stained cells were observed mainly in the epithelium of atrophied villi of small (duodenum to ileum) and large intestines (Table 2; Figure 2, panels D–F), as reported in other studies (*2*,*3*,*5*). The immunofluorescence was confined to the villous epithelial cells (Figure 2, panels D–F). A few immunofluorescence-stained cells were detected infrequently in the Peyer patches of pig 4. Lung tissues of the infected pigs did not show immunofluorescence staining, indicating that PEDV does not infect lung tissues under the conditions tested. Although PC21A strain replicated in cecum and colon epithelial cells, cellular necrosis and villous atrophy were not evident. Whether PEDV infection of the large intestine contributes to the severity of PED is unclear.

**Table 2:** Histopathologic findings in a study of the pathology of US PEDV strain PC21A in gnotobiotic pigs, 2013*

Pig status, no.	PIH at euthanasia (infection stage)	VH:CD, mean (±SD)	Antigen detection in frozen tissues†				
			Duodenum	Jejunum	Ileum	Cecum/colon	Lung
PEDV-inoculated							
1	30 (acute)	3.4 (1.7)	++	+++	+++	+++	–
2	72 (mid)	1.8 (0.3)	++	+++	+++	+++	–
3	46 (acute)	1.2 (0.3)	+	++	++	++	–
4‡	120 (later)‡	1.5 (0.2)	+	+/++	+/++	++/+++	–
5	67 (mid)	2.2 (0.4)	+	+++	+++	+	–
Negative control							
6	.	6.3 (0.7)	−	−	–	–	–

*PEDV, porcine epidemic diarrhea virus; PIH, postinoculation hour; VH:CD, ratio of villous height to crypt depth. †Detected by immunofluorescence staining. +, 1%–29% of epithelial cells showed staining; ++, 30%–59% of epithelial cells showed staining; +++, 60%–100% of epithelial cells showed staining, as described (*4*); –, no cells showed staining. ‡At 24 days of age, noninoculated pig 4 was exposed by indirect contact to pig 3 (at PIH 0) through small holes drilled into the stainless steel divider panel located between the 2 pigs in the shared pig tub isolator unit. Clinical signs and virus shedding were monitored after indirect contact. Diarrhea and vomiting developed in pig 4 approximately 2–4 h after clinical signs developed in pig 3 (i.e., in pig 4, signs developed 46–50 h after indirect contact with inoculated pig 3); pig 4 was euthanized ≈120 h after the onset of clinical signs.

All infected pigs tested at acute or later stages of infection had viral RNA titers of 4.8–7.6 log_10_ GE/mL in serum samples (Table 1). These titers were similar to those for field samples tested by real-time RT-PCR; 11 (55%) of 20 acute-phase serum samples collected from 13- to 20-week-old pigs with diarrhea from Ohio had viral RNA titers of 4.0–6.3 GE/mL. The early, severe diarrhea and vomiting and the PEDV fecal shedding at high titers may be accompanied by viremia. No infected pigs had detectable viral RNA in serum samples obtained before inoculation, and no negative control pig had detectable viral RNA during the experiment. 

## Conclusion

In 2013, the first US outbreaks of the rapidly spreading porcine virus, PEDV, caused a high number of pig deaths and substantial economic losses (*1**,**2*); however, little was known about progression of the disease. Our data confirm that US PEDV PC21A is highly enteropathogenic and acutely infects the entire intestine, but the jejunum and ileum are the primary sites of infection. PC21A infection causes severe atrophic enteritis accompanied by viremia that leads to severe diarrhea and vomiting.
